# Novel homozygous *ESAM* variants in two families with perinatal strokes showing variable neuroradiologic and clinical findings

**DOI:** 10.1038/s10038-024-01297-8

**Published:** 2024-10-17

**Authors:** Ghada M. H. Abdel-Salam, Asmaa Esmail, Dina Nagy, Sherif F. Abdel-Ghafar, Mohamed S. Abdel-Hamid

**Affiliations:** 1 Clinical Genetics Department, Human Genetics and Genome Research Institute, National Research Centre, Cairo, Egypt; 2 Medical Molecular Genetics Department, Human Genetics and Genome Research Institute, National Research Centre, Cairo, Egypt

**Keywords:** Genetics research, Clinical genetics

## Abstract

Biallelic loss of function variants in *ESAM* (endothelial cell adhesion molecule) have recently been reported in 14 individuals (9 families) presenting with prenatal intracranial hemorrhage. Here, we describe four patients from two unrelated families in whom three of them presented with variable onset encephalopathy and seizures while one only displayed profound delay without seizures. Brain MRI showed variable onset intracranial hemorrhage that evolved to hydrocephalus in 3 patients, whereas hemosiderin deposits, white matter volume loss, and porencephalic cysts were noted in one patient. Unlike the majority of described cases, the youngest brother of the first family did not show microcephaly and failure to thrive. Exome sequencing identified two novel homozygous *ESAM* variants. A splice variant (c.731-2A>G) was identified in one family which was confirmed by investigating the patient’s mRNA to result in exon skipping and early protein truncation. In addition, a missense variant (c.561G>C; p.Trp187Cys) was identified in the other family, which is the first disease causing missense variant to be described in patients with *ESAM* deficient phenotype. In addition, a maternally inherited pathogenic *MC4R* variant (c.811T>C; p.Cys271 Arg) was also identified in the youngest brother of the first family. Variants in the *MC4R* gene are associated with a non-syndromic form of obesity that could explain the unusual macrocephaly and obesity. Our work establishes *ESAM* as a tight junction gene that can present with variable neuroradiological and clinical phenotypes when mutated. Moreover, it refines the phenotype of this ultrarare syndrome and extends the number and type of variants described to date.

## Introduction

Tightjunctionopathies lead to a broad range of disease phenotypes due to abnormalities of tight junctions that play diverse roles in the integrity of vascular endothelial cells in various organs. As a consequence, *JAM2*, *JAM3* (junctional adhesion molecule 2 and 3 [MIM: 606870 and 606871]), *OCLN* (occludin [MIM: 602876] and *ESAM* (endothelial cell adhesion molecule [MIM: 614281])dysfunction can present with overlapping clinical features showing severe developmental encephalopathies and epilepsy. However, intracranial hemorrhage (ICH) seems very characteristic to *JAM3*- and *ESAM*-related phenotypes [1, 2] whereas bilateral generalized polymicrogyria and band like calcification are universal in *OCLN* [3]. In comparison, *JAM2* mutated patients usually shows severe intracranial calcification [4].

Bi-allelic loss-of-function variants in *ESAM* are assigned to a disease termed neurodevelopmental disorder with intracranial hemorrhage, seizures, and spasticity (NEDIHSS) (OMIM# 620371). This syndrome is characterized by ventriculomegaly/hydrocephalus and intracranial calcification as consequences of perinatal intracranial hemorrhage [2, 5]. So far, all reported patients harbored either stop gain, frameshift or splice variants in *ESAM* (*n* = 5 variants) [2, 5].

In this study, we describe 4 patients from two unrelated families presenting with variable neuroradiological and clinical features. We identified two homozygous variants in *ESAM* including the first missense variant supporting the pathogenic role of biallelic *ESAM* variants as the cause of a perinatal stroke.

## Subjects and methods

### Case reports

#### Family I

##### Patient 1 (I.V.1)

**Fig. 1:**
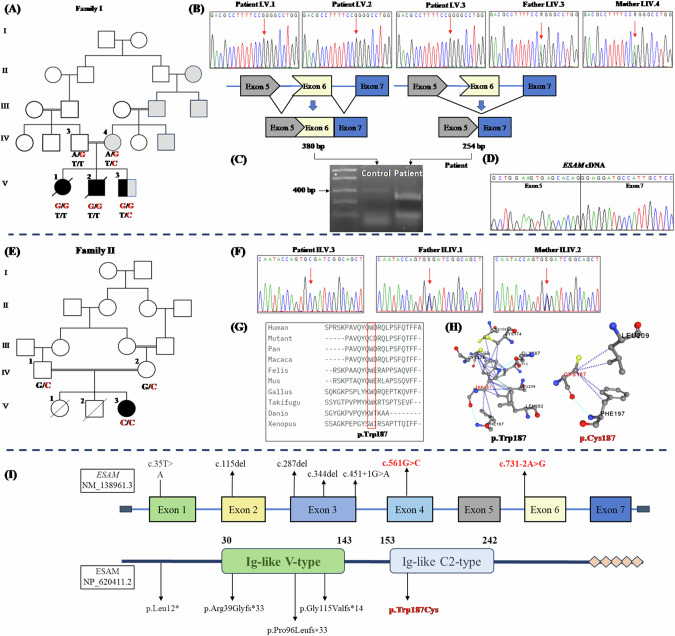
**A** Pedigree of Family I. Black filled symbols refer to individuals with *ESAM* phenotype, gray filled symbols refer to individuals with obesity, and black and gray filled symbols refers to the co-occurrence of *ESAM* phenotype and obesity in Patient I.V.3. T/T, T/C are the genotypes of the *MC4R* variant in the family. **B** Portions of the sequencing electropherograms showing the segregation of the c.731-2A>G in the family. Arrow indicates the site of variant. **C** A schematic diagram and 1% agarose gel showing partial amplification of the cDNA of the *ESAM* (from exons 4 to 7) in Patient I.V.3 and a normal control subject. Note that the patient had a shorter band in comparison to normal control. **D** Portion of the sequencing electropherograms showing skipping of exon 6. **E** Pedigree of Family II. **F** Portions of the sequencing electropherograms showing the segregation of the c.561G>C (p.Trp187Cys) in the family. Arrow indicates the site of variant. **G** The conservation of the p.Trp187 across different species. **H** The prediced 3D structure resulting from the change of p.Trp187 (wild type) to p.Cys187 (mutant) using PremPS tool. This substitution significantly alters the bonding network and the number of interacting amino acids within the protein structure. **I** Schematic diagram showing the *ESAM* and its protein domains and the location of all reported variants to date including our new variants (in red)

**Fig. 2:**
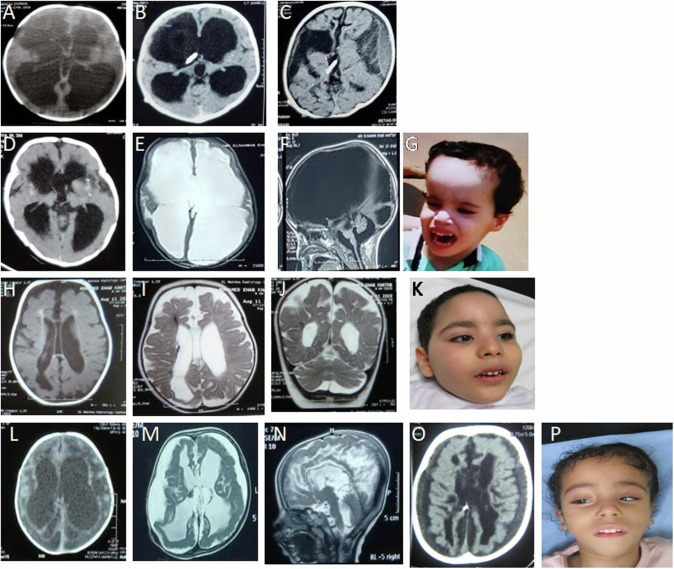
First row is for brain CT of Patient 1 at the age of 2 weeks showing multiple porencephalic cysts and severe ventriculomegaly (**A**), at the age of 4 months showing moderate ventriculomegaly and punctuate calcification after the shunt operation (**B**), and at the age of 6 months showing subdural hemorrhage (**C**). Second row is for CT of Patient 2 done at the age of 3 months and showing mild ventriculomegaly, right porencephalic cyst, intracranial calcification, and areas of encephalomalacia (**D**), at the of 4 years showing marked obstructed hydrocephalus (**E**) and infarction in the brainstem (**F**). Frontal facial photographs of Patient 2 at the age of 4 years showing macrodoilcocephaly, bitemporal narrowing and midface hypoplasia (**G**). Third row is for brain MRI of Patient 3 at the age of 4 months showing cortical and central atrophy, ventriculomegaly, cavum septum pellucidum, porencephalic cysts, hemorrhagic ischemic lesions of white matter, hemosiderin staining along the ependymal surfaces of the ventricular system, indicating previous parenchymal and intraventricular hemorrhage and mild cerebellar atrophy. **H**, **I**, **J** Frontal facial photographs of Patient 3 at the age of 3 years and 6 months showing high forehead, bitemporal narrowing, arched eyebrow and bulbous nasal tip, upturned nostrils, long eyelashes, wide nasal bridge upslanted palpebral fissures, and bow shaped mouth (**K**). Fourth row is for Patient 4. CT scan at the age of 3 weeks showing multifocal intraparenchymal hemorrhages, and diffusely hypodense brain parenchyma (**L**). Brain MRI at the age of 18 months showing subdural hemorrhage, stretched and dysmorphic corpus callosum, white matter loss, dysgyria and widened 4th ventricle stretched and atrophy of vermis and brainstem (**M**, **N**). Follow up CT scan at the age of 4 years showing increased extraaxial CSF, atrophic brain, rippled ventricles, encephalomalacia (**O**). Frontal facial photographs of Patient 4 at the age of 4½ years showing asymmetry of the skull, deep-seated eyes, squint, low-set ears, bulbous nasal tip, and bow shaped mouth (**P**)

The patient is the first child of consanguineous parents. At conception, her father was 27 years old and her mother was 24 years. No family history of a similar condition was noted. Nevertheless, the mother was obese (BMI 34.6) and she mentioned that her brother, father and grandmother had the same body build (Fig. 1A). During pregnancy the ultrasounds at the first- and second-trimester were normal whereas at 30 weeks, ventriculomegaly and enlarged biparietal diameter were noted that necessitated delivery by cesarean section (CS). Her birth weight was 3.000 kg (mean) while her birth head circumference mentioned to be large. Her birth length and Apgar score were not recorded. At the age of one week, the girl displayed bulging fontanel and a sunset appearance of eyes. She underwent shunt operation at two weeks of life. There were no complications, and she was discharged home one week after surgery. At the age of 3 months, she was admitted to a local hospital because of encephalopathy. CT scan at that time showed bilateral subdural hemorrhage (Fig. 2A–C). Later, several shunt infections with ventriculitis led to repeated shunt revisions. Comprehensive metabolic and infection work-up of blood, urine and cerebrospinal fluid (CSF) revealed no specific findings. At the age of 6 months, she developed generalized tonic-clonic seizures that were controlled on levetiracetam and valproates. The girl did not acquire any developmental milestones and was immobile and spastic with complete lack of communications. Macrocephaly, high forehead, deep seated eyes, bulbous nasal tip, and upturned nostrils were noted. She died at the age of 14 months due to unexplained thrombocytopenia and brain hemorrhage.

##### Patient 2 (I.V.2)

The pregnancy was monitored by a specialist because of the history of hydrocephalus of Patient 1. He was born by CS and without complications after 40 weeks of uneventful gestation. His birth weight was 3.28 kg (mean). His birth head circumference, length, and Apgar score were not recorded. At the age of 3 months, he had very high fever with projectile vomiting and was admitted to hospital. Serologic and biochemical investigations were normal. Brain CT showed encephalomalacia, moderate ventricular dilatation, and small calcifications located near the anterior horns of the lateral ventricles and basal ganglia (Fig. 2D–G). Until the age of 3.5 years, the boy’s head circumference was growing within the 50th percentile and the ophthalmologic examination proved to be normal. No obvious changes in the boys’ clinical status were found except for seizures that started at the age of one year in the form of focal seizures and then tonic clonic seizures and showed fair response to clonazepam and valproate. Anthropometric measures at that age showed that his weight, length, and OFC were 12.5 kg (+0.9 SD), 80 cm (+1.4 SD), and 47.5 cm (+0.7 SD), respectively. At 12 months of age, he was severely delayed. He had no clear visual fixation, and a head lag on pull to sit. Unexpectedly, at the age of 4 years the OFC crossed the 75th percentile, and his CT scan showed marked ventricular dilation with only a narrow layer of cerebral cortex on repeated neuroimaging. At that time, the boy was able to sit up with some assistance. Neurologically, he was alert but still had no clear visual fixation and never spoke. Over time, he developed lower limb spasticity. The boy displayed macrodolicocephaly with frontal bossing, deep seated eyes bulbous nasal tip, upturned nostrils and long face. He died in his sleep at the age of 5 years in the setting of several days of high fever and seizures.

##### Patient 3 (I.V.3)

He is a 3.5-year-old brother of Patients 1 and 2. He was first presented to our genetics clinic at the age of 4 months with a history of developmental delay. Due to the family history, he was born by CS at 39 weeks gestation. His birth weight was 3.37 kg (mean). He was admitted to NICU for 4 days for resuscitation because of respiratory insufficiency but details were unavailable. Initial neurologic examination at the age of 4 months was significant for axial hypotonia, appendicular hypertonia, and hyperreflexia. At that age, he had limited head control. His weight was 7.56 kg (+1 SD), length 65 cm (+1 SD) and OFC 42.5 (mean). Brain MRI revealed mild cortical and central atrophy, mild ventriculomegaly, cavum septum pellucidum, porencephalic cysts, hemorrahgic ischemic lesions of white matter, hemosiderin staining along the ependymal surfaces of the ventricular system, indicating previous parenchymal and intraventricular hemorrhage and mild cerebellar atrophy (Fig. 2H–K).

His most recent examination at the age 3½ years was significant for spastic quadriplegia and axial hypotonia, hyperreflexia of upper and lower extremities and positive Babinski sign. All developmental milestones were significantly delayed. At that age, he was not able to sit without support, could not recognize his parents and could not track objects and faces. He could make vocalizations but never had any meaningful words. His weight was 23 kg (+3 SD), length 114 cm (+ 3 SD), BMI 17.8 and OFC 51.5 (mean). His body build was very different from that of his siblings. Because of his overgrowth, bone age was arranged and showed normal results. The boy had high forehead, bitemporal narrowing, arched eyebrow and bulbous nasal tip, upturned nostrils, long eyelashes, wide nasal bridge upslanted palpebral fissures, and bow shaped mouth. No history of seizures until now. Fundus examination showed bilateral optic atrophy and increased tortuosity of retinal blood vessels. No change in the brain MRI findings on repeated imaging was noted.

#### Family II

##### Patient 4 (II.V.3)

A two-year-old Egyptian female patient is the third offspring of healthy consanguineous parents (Fig. 1E). She was born at term by CS because of hydrocephalus that was detected at the 27^th^ week of gestation by prenatal ultrasound. This pregnancy was preceded by a male baby born at term and died at 1st day of life of unknown reason and a terminated pregnancy at the 24^th^ week of gestation because of multiple congenital anomalies. The birth weight of Patient 4 was 3.000 Kg (mean). Her birth head circumference mentioned to be large. Birth length and Apgar score were not recorded. Brain CT revealed multifocal intraparenchymal hemorrhages, and diffusely hypodense brain parenchyma (Fig. 2L). Ventriculoperitoneal shunt was arranged at the age of 5 months. Generalized tonic-clonic seizures started at the age of 12 months and were controlled on levetiracetam. EEG showed multi-focal epileptogenic dysfunction. At the age of 18 months, the girl displayed irritability, crying, and projectile vomiting. At that time, her brain MRI showed subdural hemorrhage, stretched and dysmorphic corpus callosum, white matter loss, dysgyria and widened 4^th^ ventricle stretched and atrophy of vermis and brainstem (Fig. 2M, N). Platelets count and work-up for bleeding diathesis showed normal results.

We examined the case at the age of 4½ years. Her weight was 9.4 kg (–4.0 SD), length was 84 cm (–4.5 SD) and OFC was 45 cm (–5.0 SD). The girl had axial hypotonia and severe muscle wasting. There was generalized appendicular hypertonia with spasticity. Deep tendon reflexes were exaggerated. Asymmetry of the skull, deep-seated eyes, squint, low-set ears, bulbous nasal tip, bow shaped mouth and high arched palate were noted (Fig. 2P). The girl showed profound global developmental delay with very poor eye contact. Fundus examination revealed bilateral tortuous blood vessels and pale optic discs. Abdominal ultrasound and kidney function tests were normal. Follow up brain CT showed increased extraaxial CSF, atrophic brain, rippled ventricles, and encephalomalacia (Fig. 2O).

## Methods

Written informed consents were obtained from the parents, and the study was approved by the Medical Research Ethics Committee of the National Research Center, Cairo. Genomic DNA was extracted from peripheral blood samples using a standard procedure. Exome sequencing was performed for one patient from each family (Patients 3 and 4) using the SureSelect Human All Exome 50 Mb Kit (Agilent, Santa Clara, CA, USA) and analyzed on Illumina NovaSeq 6000 (Illumina, San Diego, CA, USA). The obtained sequences were aligned to UCSC human genome GRCh37/hg19 and variants were verified through the GATK pipeline. Annotation of variants was done using BaseSpace Variant Interpreter Server. We focused on rare variants (new or ≤0.001 in gnomeAD) related to the patient’s phenotype. Segregation analysis of the causative variants in the two families was performed using Sanger sequencing. To study the effect of the new splice variant identified in Family 1, total RNA was extracted from Patient 3 leukocytes followed by cDNA synsthesis and sequencing (Supplementary Methods).

## Results

Exome sequencing revealed homozygous variants in *ESAM* (NM_138961.3) in the two families. A new variant in the splice acceptor site of exon 6 was identified in Family I, c.731-2A>G. This variant resides in a homozygous region of 19.97 Mb (Supplementary Fig. 1). According to ACMG guidlines it is classified as Pathogenic (PVS1, PS3, PP1, PM2, PP3). Segregation analysis confirmed the presence of the c.731-2 A>G in the homozygous state in Patients 1 and 2 and both parents were heterozygoutes (Fig. 1B). To gain more confidence on its pathogencity, we further investagiged the patient’s mRNA and confirmed that the variant resulted in skipping of exon 6 (Fig. 1C, D) leading to a frameshift effect and early protein truncation (p.Glu287Argfs*44). In addition to the *ESAM* variant, Patient 3 was found to habrour a previosuly reported heterozyogus variant in *MC4R* (NM_005912.2), c.811T>C (p.Cys271Arg) [6]. *MC4R* variants are associated with both dominant and recessive monogenic obesity syndrome (OMIM# 618406). The variant was confirmed to be maternaly inherited by sequence analysis and was not found in the two elder affected sibs (Supplementary Fig. 2).

In Family II, a new missense variant in exon 4 was identified, c.561G>C (p.Trp187Cys). Both parents were confirmed to be carriers of the identified variant (Fig. 1F). This variant resulted from the substitution of the highly convered tryptophan at position 187 by cysteince (Fig. 1G). This variant resides in a homozygous region of 33.25 Mb (Supplementary Fig. 3). The p.Trp187Cys variant is not found in gnomAD v.4 or our inhouse database and is predicted by various bioifornmatic tools to be deleterious. Furthermore, PremPS software, which predicts the effects of missense variants on protein stability, predicts that the p.Trp187Cys leads to notable changes in the protein’s structural integrity, with a calculated ΔΔG value of 1.46 kcal/mol, indicating a destabilizing effect. Additionally, this change is expected to cause local structural rearrangements, ultimately affecting the protein’s secondary structure and increasing the risk of functional impairments, misfolding, or aggregation. According to ACMG guidlines it is classified as Variant of Uncertain Significance (PM2, PP3). The in-silico prediction scores, allele frequency, and classification of the two variants are depcticed in Supplementary Table 1.

## Discussion

To date, four neurodevelopmental disorders caused by biallelic variants in genes encoding for tight junctions have been recognized, *JAM3, JAM2, OCLN* and *ESAM*. All reported patients with *ESAM* mutated-phenotype harbored protein truncating variants that were subjected to nonsense mediated decay (NMD). In this study, we identified two new *ESAM* variants, a splice site and a missense variant extending both the number and type of *ESAM* variants (Fig. 1I). The c.731-2A>G identified in Family I was confirmed by studying the patient’s mRNA that showed exon skipping and early protein truncation and expected to escape from NMD. In Family II, a new homozygous missense variant (c.561G>C, p.Trp187Cys) was detected. This missense variant, which affects a highly conserved residue, was predicted using in-silico missense prediction tools to significantly alters the bonding network and the number of interacting amino acids within the ESAM structure. The loss of ESAM function seems to be the likely pathogenic mechanism underlying the *ESAM* mutated phenotype and the perinatal stroke observed in our two families in comparison to previously reported patients. The *ESAM* mutated patients reported here and previously emphasize that the core features are similar [2, 5]. In particular, ICH is a hallmark feature. Hydrocephalus, failure to thrive, microcephaly, seizures, retinal anomalies and profound delay are being common features in *ESAM* deficient patients (Table 1). In the meantime, ICH is also a universal feature in cases with *JAM3* that characterizes both *ESAM* and *JAM3* from other tightjunctionopathies. Based on the literature review, we suggest that ICH in *ESAM* mutated patients is often less severe. Extensive multifocal intraparenchymal and intraventricular hemorrhage evolving to white matter liquefaction, cystic encephalomalacia and subependymal calcification were common in *JAM3* deficient patients [1, 2, 5, 7, 8] whereas subdural hemorrhage, parenchymal germinal matrix intraventricular and periventricular hemorrhage were reported in *ESAM* mutated patients. However, the later shows a wide range of clinical severity ranging from severe intrauterine hemorrhage and hydrocephalus to porencephalic cyst and mild ventriculomegaly [2, 5]. The milder presentation of *ESAM* mutated patients highlights the notion about its redundant functional role in physiological angiogenesis and the presence of other endothelial adhesion molecules which may compensate for its dysfunction [2].

**Table 1:** The demographic, clinical, and genetic data of patients with biallelic *ESAM* variants

	This study				Alshammari et al. [5]	Lecca et al. [2]
	Patient 1(I.V.1)	Patient 2 (I.V.2)	Patient 3 (I.V.3)	Patient 4 (II.V.3)	Family 3 (III:5)	
Gender	F	M	M	F	M	8 M/5 F/ 1 unknown
Age at examination	4 m	1 y	3½y	4½y	3 y	From 13 m to 13 y
Ethnicity	Egyptian				NA	Turkish, Algerian, Spanish, Arab Bedouin
Consanguinity	+			+	+	12/14
Prenatal hydrocephalus	+	–	–	+	NA	7/13
Weight in kg (z score)	5.9 (mean)	12.5 ( + 0.9)	23 ( + 3)	9.5 (-4)	NA	7/9 <10th percentile
Height in cm (z score)	60 (mean)	80 ( + 1.4)	114 ( + 3)	84 (-4.5)	NA	8/8 <10th percentile
OFC in cm (z score)	41 (mean)	47.5 ( + 0.7)	51.5 (mean)	45 (-5)	NA	4/9 <10th percentile
**Neurological examination**						
Hypertonia or spasticity	+	+	+	+	+	Spasticity (9/9)
Exaggerated reflexes	+	+	+	+	+	+ (9/9)
Degree of developmental quotient	Severe	Severe	Severe	Severe	Severe	Severe (9/9)
**Seizures**	+	+	–	+	+	+ (9/9)
Onset	6 m	1 y	–	1 y	NA	NA
Type	GTC	Focal, GTC	–	GTC	NA	Focal, GTC, myoclonic
Status epilepticus	+	-	–	–	NA	NA
Response to AEDs	fair	fair	–	fair	NA	NA
Abnormal EEG	NA	Normal	Normal	Normal	NA	(2/9) showed hypsarrhythmia
**Imaging abnormalities**						
Age at neuroimaging	2 weeks, 3 m, 6 m		4 m, 3 y 6 m	3 weeks, 3 m, 1½y, 4 y	4 y	Prenatal and first months of life
Porencephaly	+	+	+	+	+	+ (3/9)
Leukomalacia	+	+	+	+	+	+ (3/9)
abnormal myelination	+	+	+	+	+	+ (5/9)
Intracranial hemorrhage	+	+	+	+	Chronic intraventricular hemorrhage and hemorrhagic periventricular microcystic lesions	+ (9/9)
Ventriculomegaly	+	+ Severe	+ Mild	+	+ Mild	+ (9/9)
Hydrocephalus	+	+ Progressive	–	+	–	+ (7/9)
Intracranial calcification	+	+	+	+	–	+ (8/9)
Progressive ICH	+	+	–	+	–	–
Fundus examination	NA	Normal	Pale optic disc, increased tortuosity of retinal blood vessels	Pale optic disc, increased tortuosity of retinal blood vessels	Severe strabismus	4/9 (retinal ischemia, increased tortuosity of retinal blood vessels, retinal hemorrhage)
Facial features	Macrocephaly, high forehead, deep seated eyes bulbous nasal tip, upturned nostrils	Macrodolicocephaly with frontal bossing, deep seated eyes, bulbous nasal tip, upturned nostrils, and long face	High forehead, bitemporal narrowing, arched eyebrow and bulbous nasal tip, upturned nostrils, long eyelashes, wide nasal bridge upslanted palpebral fissures, and bow shaped mouth	Asymmetry of head, high forehead, bulbous nasal tip, bow shaped mouth and high arched palate	NA	Bitemporal narrowing (6/9), highly arched eyebrow and bulbous nasal tip (5/9), long eyelashes (4/9), abnormal vermilion (4/9), high narrow palate (3/9), wide nasal bridge (3/9), upslanted palpebral fissures (3/9), and microretrognathia (2/9)
Age of death	15 m	5 y	Alive	Alive	NA	2/9 (at 5 y and 15 m) and 3 terminations of pregnancy and a still birth
***ESAM*** **variant** (NM_138961.3)	c.731-2A>G			c.561G>C (p.Trp187Cys)	c.344del (p.Gly115Valfs*14)	c.35T>A (p.Leu12*), c.115del (p.Arg39Glyfs*33), c.287del (p.Pro96Leufs*33), c.451+1G>A

*AEDs* antiepileptic drugs, *EEG* electroencephalogram, *F* female, *GTC* generalized tonic-clonic, *ICH* intracranial hemorrhage, *M* male, *m* month, *NA* not available, *y* year

Our report shows intrafamilial and interfamilial phenotypic differences. Of interest, Patient 1 and 4 had identical clinical course of congenital hydrocephalus and intracranial calcifications, then they developed peculiar severe subdural hemorrhage which only subsequently appeared at the age of 6 months and 1½ year, respectively. In comparison, Patient 2 developed postnatal hydrocephalus that showed progressive course whereas Patient 3 only had porencephaly and diffuse hemosiderin deposits. Interestingly, Patient 4 and 1 harbored different types of variants, a missense and a splice variant, respectively. Therefore, a correlation between the variant type and the overall severity of the patients’ phenotypic manifestations could not be speculated.

Our findings are of significance in understanding the pathogenesis of *ESAM* since it suggests variable tissue sensitivity to the underlying, and currently undefined, disease associated cellular perturbation. It will be interesting to monitor the neuroradiological follow-up of these patients. The question remains as to whether such patients are at risk of repeated ICH over time, and/or at what age one might be reassuring that ICH will not develop. Identification of further cases will help to better refine the phenotypic spectrum and delineate the neuroradiological characteristic aspects of this complex disorder.

Our patients and those described previously share similar dysmorphic facial features (including bitemporal narrowing, triangular face, smooth philtrum, high-arched eyebrows, bulbous nasal tip, long eyelashes, abnormal vermilion, high narrow palate, wide nasal bridge, upslanted palpebral fissures, prominent chin, and microretrognathia) (Table 1) although non-specific [2].

Given the essential role of *ESAM* in angiogenesis, endothelial permeability, tube formation, and vascular density, it is not surprising to have abnormal retinal and renal vascular morphology in 50% (5/10) and 11% (1/9), respectively [2, 5]. Patients 3 and 4 showed only abnormal retinal vascular morphology.

Unexpectedly, the growth curve of Patient 3 showed childhood obesity. However, such obesity has never been reported in patients with disease-causing variants of *ESAM*. Obesity could be explained by the identified heterozygous missense variant in *MC4R* which is the most common genetic cause of obesity [6] and missense variants are the most prevalent [6, 9].

In conclusion, we contribute to the delineation of *ESAM* -related tightjunctionopathies, highlighting the inter and intrafamilial variability and the course of this condition. Our findings strongly indicate that other genetic and/or non-genetic factors may modulate the progression and expression of the patients’ phenotypes.

## Supplementary information

Supplementary Figures
