# Supplementary material for: Novel homozygous ESAM variants in two families with perinatal strokes showing variable neuroradiologic and clinical findings
Source: J Hum Genet. 2024 Oct 17;70(2):67–74. doi: 10.1038/s10038-024-01297-8 (PMC11762408; doi:10.1038/s10038-024-01297-8)
Supplement: Supplementary file 1 — Supplementary Figures [file 10038_2024_1297_MOESM1_ESM.doc]

**Supplementary Methods**

**Segregation of the *ESAM* variant**

The c.731-2A>G variant in *ESAM* gene (NM_138961.3) identified by exome sequence was confirmed in the parents and the other two affected siblings using Sanger sequencing. The region encompassing this variant (intron 5/exon 6) was amplified using the following primers sequence: Forward: 5’-CCACCCCTCTTCTTGACAGG-3’ and Reverse: 5’-CCACTGACCCCATCCCAATA-3’. Primers were designed by Primer3 SOFTWARE. PCR cycling conditions were: initial denaturation at 96°C for 5 min; 30 cycles of denaturation at 96°C for 30 sec; annealing at 62°C for 30 sec; extension at 72°C for 30 sec, and a final extension at 72°C for 5 min. PCR products were purified using Exo-SAP PCR Clean-up kit (Fermentas, Germany) and sequenced in both directions using the BigDye Terminator v3.1 Cycle Sequencing Kit (Applied Biosystems, Foster City, CA, USA) and analyzed on the ABI Prism 3500 Genetic Analyzer (Applied Biosystems) according to manufacturer's instructions.

**Functional study of the c.731-2A>G variant**

To study the effect of newly identified *ESAM* variant (c.731-2A>G) on splicing, total RNA was extracted from the patient’s leukocytes using QIAamp RNA Blood Mini Kit (Qiagen, Germany). Five µg of total RNA were reverse transcribed into cDNA using QuantiTect Reverse Transcription Kit (Qiagen, Germany). The synthesized cDNA was then used as a template for partial amplification of the *ESAM* gene (from exons 4 to 7) using one pair of primers: 5’-ATCGGCAGCTTCCATCCTT -3’ and 5’-AGGTGACAGAGGAAAGGGTC -3’ under the following conditions: 96°C for 5 min, a total of 30 cycles of 96°C for 30 sec, annealing at 62.5°C for 30 sec, 72°C for 30 sec, and a final extension of 72°C for 5 min. PCR products were separated by 1% agarose gel electrophoresis and then purified and sequenced as described above.

**Segregation of the *MC4R* variant**

The c.811T>C (p.Cys271Arg) variant in *MC4R* (NM_005912.2) gene identified by exome sequence was confirmed in mother using Sanger sequencing. Exon 1 harboring this variant was partially amplified using the following primers sequence: Forward: 5’-GTCCACATGTTCCTGATGGC -3’ and Reverse: 5’-ATATTGCGTGCTCTGTCCCC-3’. Primers were also designed by Primer3 SOFTWARE. PCR cycling conditions, and sequencing methods are the same as described above.

**Supplementary Figures**

**
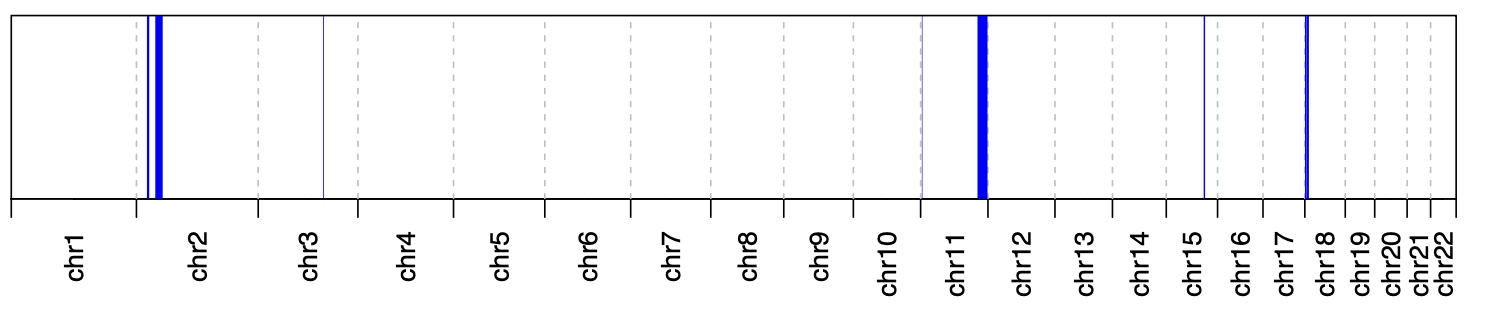
**

**Supplementary Fig. 1**: Homozygosity mapping of Patient 3 using automap tool showing a homozygous stretch of 19.97 Mb starting from chr11: 114,276,552 to chr11:134,241,632.

**Supplementary Fig. 2**: Portions of the sequencing electropherograms showing the detection of the *MC4R* variant in the mother while the father, Patients 1, and 2 were wild type. Arrow indicates site of the variant.

**
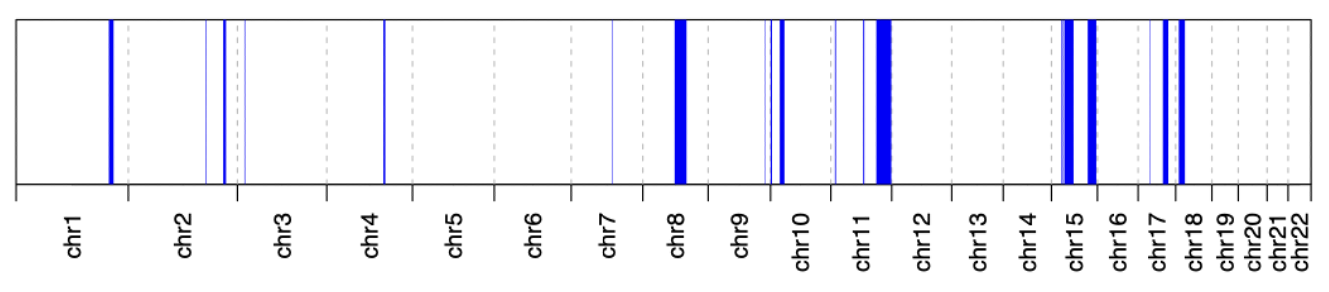
**

**Supplementary Fig. 3**: Homozygosity mapping of Patient 4 using automap tool showing a homozygous stretch of 33.25 Mb starting from chr11: 100,998,623 to chr11: 134,244,123.

**Supplementary Tables**

**Supplementary Table 1:** In silico prediction scores, population frequency, and conservation score of the identified*ESAM*variants

|  | **Variant in *ESAM***(NM_138961.3)(nucleotide change, amino acid change, exon) | |
| --- | --- | --- |
|  | **c.561G>C**  **p.Trp187Cys**  **Exon: 4** | **c.731-2A>G**  **Intron5/Exon 6** |
| **PolyPhen** | Probably damaging(0.989) | - |
| **SIFT** | Deleterious (0) | - |
| **REVEL** | Supporting (0.584) | - |
| **CADD** | 25.9 | 33 |
| **MetaRNN** | Pathogenic Strong (0.9806) | - |
| **BayesDel addAF** | Pathogenic Moderate (0.3143) | Uncertain(0.1524) |
| **Mutation Taster** | Disease causing(1) | Disease causing(1) |
| **AlphaMissense** | Pathogenic Strong (0.9989) | - |
| **EIGEN** | Pathogenic Moderate(0.9239) | Pathogenic Moderate (0.9499) |
| **Mutation assessor** | Pathogenic Moderate (4.7) | - |
| **MutPred** | Pathogenic Moderate (0.85) | - |
| **PROVEAN** | Pathogenic Moderate (-12.5) | - |
| **LRT** | Pathogenic Supporting (0) | - |
| **MetaRNN** | Damaging (0.9804) | - |
| **DEOGEN2** | Damaging(0.96) | - |
| **dbscSNV** | - | Pathogenic Strong ( 0.9999) |
| **MaxEntScan** | - | Pathogenic Strong (7.9549) |
| **Splice AI**  **Acceptor gain delta score**  **Acceptor loss delta score** | - | 0.85  0.98 |
|  |  |  |
| **ACMG classification** | Variant of Uncertain Significance  PM2, PP3 | Pathogenic  PVS1, PS3, PP1, PM2, PP3 |
| **gnomAD v.4 exome allele frequency** | Not reported | Not reported |
| **Conservation Scores phyloP100** | 4.526 | 4.779 |
